# Pegylated recombinant human granulocyte colony-stimulating factor enhances radiotherapy completion and safety in concurrent chemoradiotherapy for cervical cancer: a retrospective analysis

**DOI:** 10.3389/fonc.2025.1640418

**Published:** 2025-09-04

**Authors:** Lingli Zhao, Yanhong Zhai, Luhong Meng, Yajuan Ren, Nannan Liu, Xuejiao Xing, Xin Wen, Gaoli Niu

**Affiliations:** Department of Gynecologic Oncology, First Affiliated Hospital of Henan Polytechnic University, Jiaozuo, China

**Keywords:** PEG-rhG-CSF, cervical cancer, concurrent chemoradiotherapy, radiotherapy completion, hematologic toxicity

## Abstract

**Objective:**

To evaluate the impact of pegylated recombinant human granulocyte colony-stimulating factor (PEG-rhG-CSF) on radiotherapy completion rates and safety in cervical cancer patients undergoing concurrent chemoradiotherapy.

**Methods:**

This retrospective study analyzed 60 cervical cancer patients treated at the researchers’ hospital between October 2021 and October 2024. The patients were divided into two groups: the PEG-rhG-CSF group (n = 30), which received pegylated recombinant human granulocyte colony-stimulating factor (PEG-rhG-CSF), and the non-PEG-rhG-CSF group (n = 30), which received recombinant human granulocyte colony-stimulating factor (rhG-CSF). Key outcomes, including radiotherapy completion time, hematological toxicity, and non-hematological adverse events, were compared between the two groups.

**Results:**

The results demonstrated that 83.33% of patients in the PEG-rhG-CSF group completed radiotherapy within 8 weeks, compared to 51.72% in the non-PEG-rhG-CSF group. The time to bone marrow suppression was significantly longer in the PEG-rhG-CSF group (4.93 ± 0.22 weeks) than in the non-PEG-rhG-CSF group (4.07 ± 0.22 weeks). Additionally, the lowest white blood cell (WBC) count in the PEG-rhG-CSF group was 2.46 ± 0.13 × 10^9^/L, which was significantly higher than 2.04 ± 0.13 × 10^9^/L in the non-PEG-rhG-CSF group (*P* = 0.025). The incidence of grade 3/4 neutropenia was significantly lower in the PEG-rhG-CSF group (16.67%) compared to the non-PEG-rhG-CSF group (56.67%) (*P* = 0.003). Furthermore, no febrile neutropenia (FN) cases were observed in the PEG-rhG-CSF group, whereas six cases occurred in the non-PEG-rhG-CSF group (*P* < 0.05). Non-hematologic adverse reactions were comparable between the two groups, and the bone pain associated with PEG-rhG-CSF was tolerable.

**Conclusion:**

The study demonstrates that PEG-rhG-CSF has significantly improved radiotherapy completion rates and reduced the incidence of grade 3/4 leukopenia and neutropenia in cervical cancer patients undergoing concurrent chemoradiotherapy. Additionally, PEG-rhG-CSF has exhibited a favorable safety profile, with manageable adverse effects, making it a promising supportive treatment option in this setting.

**Synopsis:**

This study explores the application of PEG-rhG-CSF in concurrent chemoradiotherapy for cervical cancer. A retrospective analysis evaluates its effectiveness in helping patients’ complete radiotherapy on time, reducing hematologic toxicity, and ensuring safety.

## 1 Introduction

Cervical cancer is the fourth most common cancer among women worldwide. According to the latest cancer data released jointly by China and the United States in 2022, there are approximately 150,700 new cases of cervical cancer in China each year, resulting in about 55,700 deaths (1–4). It is known that human papillomavirus (HPV) is the primary pathogenic factor for cervical cancer. Although cervical cancer can be prevented through vaccination with HPV vaccines, many patients are still diagnosed at an advanced stage. Compared to early-stage diagnosis, the prognosis for advanced-stage cervical cancer is poorer, with a 5-year survival rate of 20% (5–8). The current treatment for cervical cancer mainly involves surgery, radiotherapy, and chemotherapy, with surgery being the primary option in the early stages and radiotherapy in the advanced stages, all aimed at achieving a curative outcome (9–12). According to the National Comprehensive Cancer Network (NCCN) guidelines, for certain stages IB3, IIA2, and IIB-IVA cervical cancer, the preferred initial treatment can be concurrent chemoradiation plus brachytherapy with or without induction chemotherapy, and it is most effective when completed within 8 weeks. Neoadjuvant chemotherapy is also an option, and after treatment, if the lesion persists or progresses, systemic therapy can be considered (13–16). Some studies have found that, compared with the same dose of radiotherapy, concurrent chemotherapy can increase the overall survival rate of cervical cancer patients by 6% (17, 18). However, with the accumulation of radiation doses and chemotherapy drugs, adverse reactions such as reduced leukocytes and neutrophils, anemia, thrombocytopenia, and gastrointestinal issues may occur (17, 19). When grade 3/4 leukopenia and neutropenia occur, the current primary treatment measures involve suspending radiotherapy and administering recombinant human granulocyte colony-stimulating factor (rhG-CSF) via daily subcutaneous injection. This approach often results in reduced radiotherapy doses or prolonged treatment cycles for some patients, ultimately leading to poor prognosis, increased risk of recurrence, and diminished quality of life, among other adverse outcomes (20).

RhG-CSF is a rapidly acting G-CSF that effectively reduces the risk of neutropenia and febrile neutropenia (FN) following chemotherapy (21). However, due to its short half-life, it requires frequent injections and monitoring of blood cells, which not only increases the risk of infection but also affects quality of life and treatment compliance (22). Polyethylene glycol-conjugated recombinant human granulocyte colony-stimulating factor (PEG-rhG-CSF) is a glycoprotein formed by the covalent conjugation of PEG and rhG-CSF. It acts on hematopoietic cells and can bind to specific receptors on the surface of granulocyte progenitor cells, thereby promoting the proliferation, differentiation, and maturation of granulocyte progenitor cells (23, 24).

PEG-rhG-CSF exerts its therapeutic effects through multiple biological mechanisms. By extending its half-life, it provides sustained activation of G-CSF receptors in bone marrow (25), promoting proliferation and differentiation of granulocyte colony-forming units (CFU-Gs) and significantly increasing absolute neutrophil counts (ANC). Pharmacokinetic studies demonstrate dose-dependent peak concentrations, with optimal efficacy when administered 48 hours post-chemotherapy. The drug operates through a biphasic mechanism: initially mobilizing mature CD11b^+^ Ly6G^+^ neutrophils into peripheral circulation within 12 – 24 hours (26), followed by CXCR4 upregulation that enhances hematopoietic stem cell homing and differentiation, maintaining ANC reaches the normal range for 3 – 5 additional days compared to conventional rhG-CSF (27). PEG-rhG-CSF specifically modulates neutrophil subsets, increasing the proportion of functional CD16^hi^ CD62L^lo^ mature subsets (to 70 - 80% of total neutrophils) while reducing immunosuppressive CD16^lo^ CD62L^hi^ populations, resulting in 40% reduction in febrile neutropenia incidence (28). Its anti-inflammatory effects include ≥50% reduction in CRP and PCT levels, shortening fever duration by 2.3 days (29), and decreasing chemotherapy-induced tissue damage markers (GST/ALT), with animal models showing 60% reduction in hepatic necrosis area (30). These protective effects correlate with ANC recovery. However, high doses (>100μg/kg) may transiently elevate IL - 6 levels, requiring monitoring for bone pain and other potential adverse effects (30). Collectively, PEG-rhG-CSF’s multifaceted actions - enhancing granulopoiesis, optimizing neutrophil function, mitigating immunosuppression, and controlling inflammatory responses - make it an effective option for managing chemotherapy-induced neutropenia while providing additional immunomodulatory benefits.

Compared to rhG-CSF, which requires daily injections and continuous use, PEG-rhG-CSF has a half-life of 42 – 62 hours, making it a long-acting and self-regulating form of rhG-CSF (31). It can be administered subcutaneously once every 21 days, which not only avoids the pain associated with frequent injections but also helps maintain a relatively stable level of white blood cells and neutrophils, reducing the risk of insufficient radiation doses or extended radiation cycles due to decreases in white blood cells and neutrophils (32). Nowadays, PEG-rhG-CSF has been widely used in the treatment of cervical cancer, lung cancer, breast cancer, and hematological malignancies for radiation and chemotherapy-induced leukopenia and neutropenia (33–35).

Currently, research on the application of PEG-rhG-CSF in concurrent chemoradiotherapy for cervical cancer primarily focuses on its role in mitigating neutropenia. According to the NCCN guidelines, treatment should be completed within 8 weeks to achieve optimal efficacy. However, due to bone marrow suppression caused by concurrent chemoradiotherapy, patients often experience varying degrees of myelosuppression during this period, leading to prolonged radiotherapy cycles (20). Research on the role of PEG-rhG-CSF in improving radiotherapy completion rates during concurrent chemoradiotherapy for cervical cancer remains relatively limited. This study employs a retrospective analysis to evaluate the application value of PEG-rhG-CSF in facilitating the completion of radiotherapy within 8 weeks, reducing hematologic toxicity, and assessing its safety profile.

## 2 Material and methods

### 2.1 Patients and inclusion criteria

We conducted a retrospective analysis of data from 60 patients with cervical cancer who underwent definitive concurrent chemoradiotherapy at the researchers’ hospital from October 2021 to October 2024. Inclusion criteria: (1) Patients with cervical cancer staged as IB3, IIA2, and IIB-IVA according to FIGO 2018, who were jointly examined by two attending Gynecologist physicians; (2) All pathologically confirmed to have squamous cell carcinoma, with differentiation grades of G2, G3, or G2 - 3; (3) Treatment plan consists of concurrent chemoradiotherapy plus brachytherapy with or without adjuvant chemotherapy; (4) Prior to radiotherapy, white blood cell count (WBC) > 4.0 × 10^9^/L, and absolute neutrophil count (ANC) > 2.0 × 10^9^/L; (5) Age between 18 and 70 years old, ECOG performance status ≤ 2; (6) Patients who have not received prior chemotherapy/surgery/targeted therapy, with no history of previous malignant tumors, and no severe hematological, immune system, or other systemic diseases; (7) Complete and traceable imaging data.

A total of 60 patients were enrolled based on the inclusion criteria and divided into two groups according to whether they received regular subcutaneous injections of PEG-rhG-CSF during treatment: the PEG-rhG-CSF group (30 patients) received 6 mg of PEG-rhG-CSF via subcutaneous injection every 21 days, while the non-PEG-rhG-CSF group (30 patients) received subcutaneous injections of rhG-CSF only when a decrease in WBC or ANC was observed during treatment.

### 2.2 Chemoradiotherapy protocol

During radical radiotherapy, external-beam irradiation is delivered using intensity-modulated radiation therapy (IMRT) at a total dose of 45 Gy in 25 fractions of 1.8 Gy each. Positive nodal regions receive an additional boost to 50 Gy. Patients with parametrial involvement or pelvic sidewall invasion receive a parametrial boost of 10 – 12 Gy administered in 5 – 6 fractions of 2.0 Gy each. Intracavitary brachytherapy is generally initiated after approximately 20 fractions of external-beam radiotherapy (weeks 4 – 5), when substantial tumor regression and restoration of pelvic anatomy facilitate applicator insertion and reduce bleeding risk. During external-beam therapy, one 6-Gy fraction is delivered weekly (with EBRT omitted on that day). After completion of EBRT, the frequency of brachytherapy is increased to twice weekly. A total of five 6-Gy fractions are administered using an intrauterine tandem applicator, with interstitial techniques utilized when necessary. Pelvic MRI and gynecological examinations are subsequently performed to assess residual lesions. If active disease persists, an extra application of brachytherapy dose of 3 – 6 Gy may be administered within the tolerance of critical organs, ensuring the entire treatment course is completed within 8 weeks. The therapeutic goal is to achieve an equivalent dose in 2-Gy fractions (EQD_2_) of 85 – 92 Gy to 90% of the high-risk clinical target volume (HRCTV D90).

Target volumes and organs at risk (OARs) were precisely delineated using CT-based multi-modal image fusion: the HR-CTV encompassed primary lesions and positive lymph nodes, while the IR-CTV covered potential areas of microscopic spread. OARs included the bladder, rectum, small bowel, and bone marrow cavity, all with predefined dose constraints. Dose-volume histograms (DVH) of the bone marrow cavity were utilized to assess the risks of myelosuppression, aiming to achieve an optimal balance between tumor control and bone marrow protection.

During radiation therapy, cisplatin at a dose of 40 mg/m² is administered as single-agent concurrent chemotherapy for 4 to 6 cycles (13, 16). Four weeks after the completion of radiotherapy, a re-evaluation is conducted with imaging studies, either an enhanced abdominal CT or an enhanced pelvic MRI, to determine whether to administer adjuvant chemotherapy. If there is still active disease present, a chemotherapy regimen combining cisplatin with paclitaxel will be given for 3 to 4 cycles every 21 days.

### 2.3 Management of myelosuppression

The 60 patients were divided into two groups based on whether they received regular subcutaneous injections of PEG-rhG-CSF during treatment. Among them, 30 patients were administered a single 6 mg subcutaneous dose of PEG-rhG-CSF within 24 to 48 hours before the first radiotherapy session, with subsequent doses repeated every 21 days thereafter (36). For patients who experienced bone pain or muscle pain, non-steroidal anti-inflammatory drugs (NSAIDs), such as ibuprofen sustained-release capsules or loxoprofen sodium tablets, were administered for 3 to 5 days during treatment (10). A complete blood count was performed every Friday, and all treatment-emergent adverse events were recorded and graded according to the Common Terminology Criteria for Adverse Events (CTCAE, version 5.0) (37). The threshold for supplemental rhG-CSF administration in this study was determined based on our institutional laboratory reference ranges and clinical experience, taking into full consideration patients’ underlying conditions, the risk of myelosuppression during radiotherapy, and the high requirement for uninterrupted treatment. Compared with some guideline recommendations, this threshold is more stringent and was adopted to maximize patient safety and to ensure the smooth progression of radiotherapy. If the WBC count was below 3.5 × 10^9^/L or the ANC was below 1.8 × 10^9^/L, rhG-CSF was supplemented at a dose of 5 μg/kg/day until the WBC count exceeded 3.5 × 10^9^/L and the ANC exceeded 1.8 × 10^9^/L. In the non-PEG-rhG-CSF group, 30 patients also underwent weekly complete blood count checks every Friday. If either the WBC or ANC fell below these thresholds during treatment, subcutaneous rhG-CSF was administered immediately as an intervention.

### 2.4 Study endpoints

The primary endpoints include the radiotherapy completion rate within 8 weeks, the incidence of grade 3/4 neutropenia, and the incidence of FN. The secondary endpoints include the time of onset of neutropenia and treatment safety. Additionally, routine blood tests are conducted weekly during radiotherapy to evaluate WBC count, ANC, hemoglobin (HB) levels, and platelet (PLT) counts, with comparative analysis performed. Failure to complete radiotherapy within 8 weeks is defined as a delay, and treatment will be paused in the event of grade 3/4 toxicity.

### 2.5 Statistics analysis

Data analysis was conducted using GraphPad Prism version 10 software. If the data follows a normal distribution, a t-test was used to analyze the measured data, which is expressed as the mean ± standard deviation. For categorical data, Chi-square or Fisher’s exact test was used, and the data is presented as percentages. A *p*-value of < 0.05 indicates that the difference is statistically significant.

## 3 Results

### 3.1 Patient background

**Figure 1:**
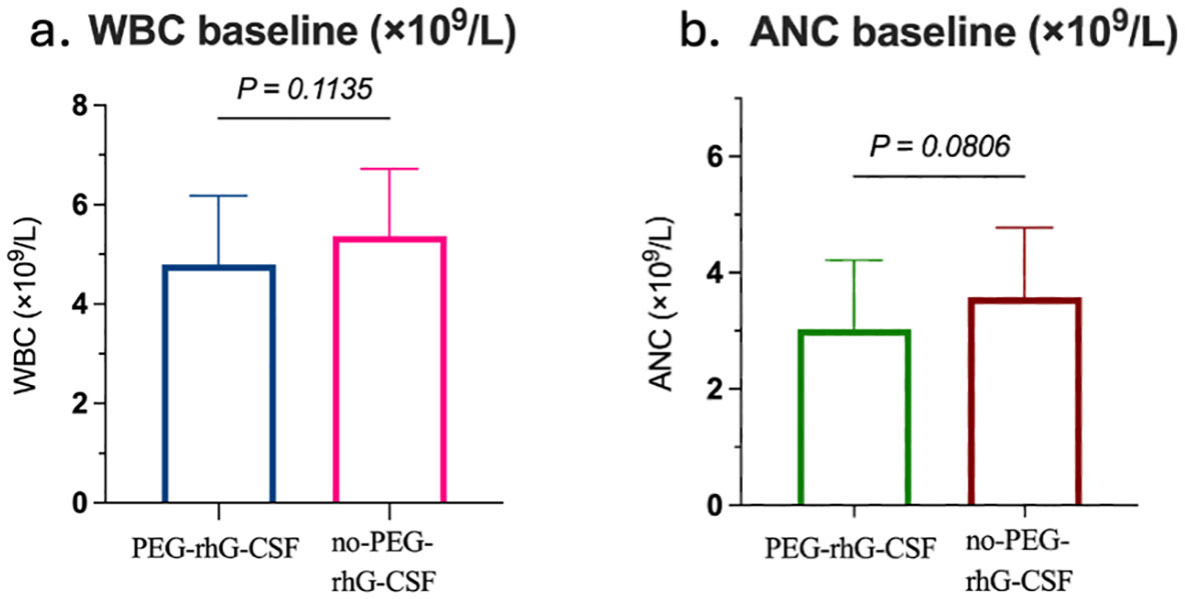
Comparison between the PEG-rhG-CSF group and the no PEG-rhG-CSF group: **(a)** The bar chart shows comparable baseline WBC, with no significant difference (*p*=0.1135). **(b)** The bar chart shows comparable baseline ANC, with no significant difference (*p*=0.0806).

The baseline characteristics of 60 patients were analyzed, and a summary of their background is presented in **Table 1**. Based on whether they received PEG-rhG-CSF during treatment, the patients were divided into two groups: the PEG-rhG-CSF group and the non-PEG-rhG-CSF group. The average ages of the PEG-rhG-CSF and non-PEG-rhG-CSF groups were 54.67 ± 1.39 years and 54.30 ± 1.57 years, respectively, with BMI values distributed as 24.36 ± 3.78 and 24.15 ± 3.06. There were no statistically significant differences between the two groups. The pathological types were all squamous cell carcinoma, with differentiation grades of G2, G3, or G2 - 3, and the differences between the two groups were not statistically significant (*P* = 0.865). According to the FIGO staging, the PEG-rhG-CSF group included 2 cases of stage IB3 and IIA2, 10 cases of stage IIB, 9 cases of stage IIIA+IIIB, and 9 cases of stage IIIC. The non-PEG-rhG-CSF group comprised 3 cases of stage IB3 and IIA2, 7 cases of stage IIB, 8 cases of stage IIIA+IIIB, and 12 cases of stage IIIC. There was no statistically significant difference in staging between the two groups (*P* = 0.809). In summary, there were no significant differences in baseline characteristics between the two groups. Furthermore, before treatment, there were no statistically significant differences in the baseline levels of WBC and ANC between the two groups, as shown in **Figure 1**.

**Table 1:** Baseline characteristics of patient.

Characteristic	PEG-rhG-CSF	Non-PEG-rhG-CSF	Statistics	*P*
Age (yr)	54.67 ± 1.39	54.30 ± 1.57	*t* = 0.175	0.862
BMI (kg/m^2^)	24.36 ± 3.78	24.15 ± 3.06	*t* = 0.228	0.820
Pathological type				
Squamous cell carcinoma	30 (100%)	30 (100%)		
Differentiation grades			*χ^2^*	0.865
G2	18 (60%)	20 (66.67%)		
G3	8 (26.67%)	7 (23.33%)		
G2 - 3	4 (13.33%)	3 (10%)		
FIGO staging			*χ^2^*	0.809
IB3, IIA2	2 (6.0%)	3 (9.0%)		
IIB	10 (30.0%)	7 (21.0%)		
IIIA+IIIB	9 (27.0%)	8 (24.0%)		
IIIC	9 (27.0%)	12 (36.0%)		

Values are presented as median (range) or number (%). BMI, Body Mass Index; G, Grade; FIGO, International Federation of Gynecology and Obstetrics.

### 3.2 The time to complete radiotherapy

**Figure 2:**
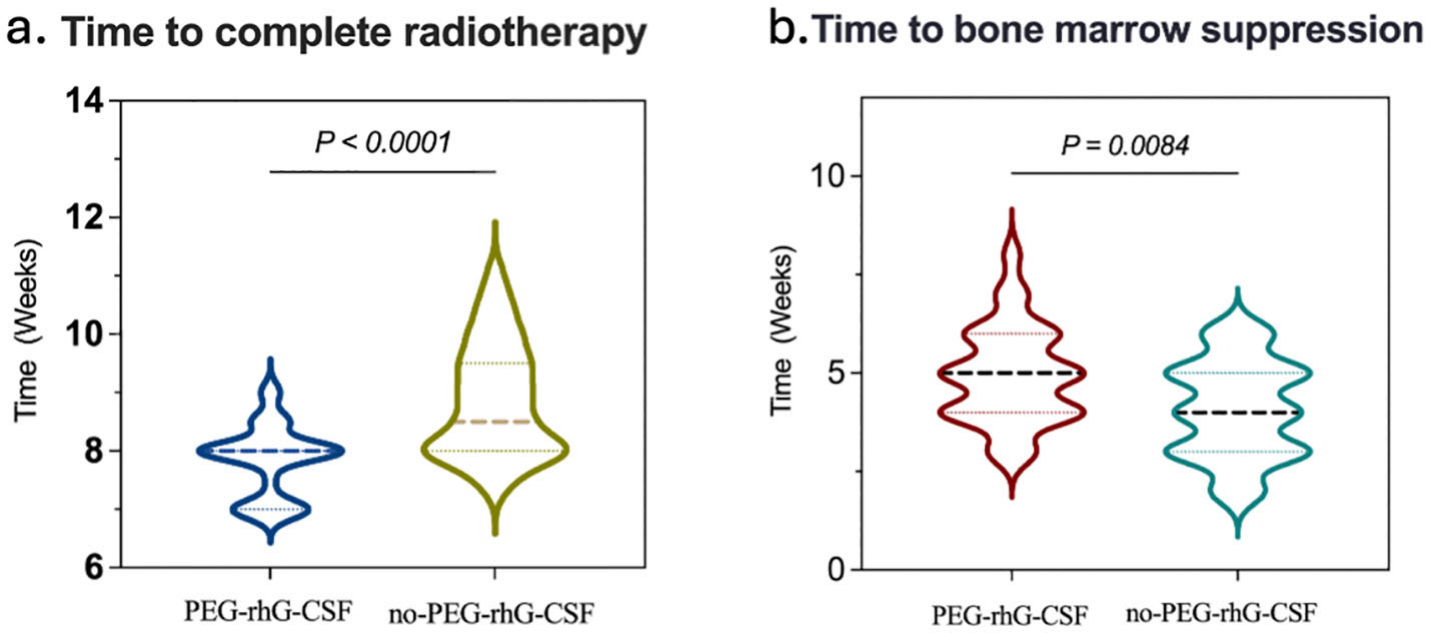
Comparison between the PEG-rhG-CSF group and the no PEG-rhG-CSF group: **(a)** The violin plot indicates a shorter time to complete radiotherapy in the PEG-rhG-CSF group (*p*<0.0001). **(b)** The violin plot indicates a delayed onset of bone marrow suppression in the PEG-rhG-CSF group (*p*=0.0084).

The equivalent biological dose (EQD2) delivered to the treatment targets was 89.25 ± 2.56 Gy in the PEG-rhG-CSF group and 88.88 ± 2.39 Gy in the non-PEG-rhG-CSF group, with no statistically significant difference between the two groups (P = 0.062) (**Table 2**). However, in terms of overall radiotherapy duration, the PEG-rhG-CSF group completed treatment in an average of 7.85 ± 0.604 weeks, which was significantly shorter than the 8.86 ± 0.972 weeks observed in the non-PEG-rhG-CSF group (**Table 2**). Furthermore, 83.33% of patients in the PEG-rhG-CSF group were able to complete radiotherapy within 8 weeks, compared to only 51.72% in the non-PEG-rhG-CSF group—a statistically significant difference (*P* < 0.001) (**Figure 2A**). These findings indicate that the use of PEG-rhG-CSF facilitates timely completion of radiotherapy and reduces the risk of treatment delays. Regarding hematological toxicity, we performed a detailed analysis of hematologic recovery following supportive care. For patients with grade 1 – 2 myelosuppression, leukocyte-elevating therapies generally resulted in blood count recovery within 3 – 5 days. For grade 3 – 4 myelosuppression, radiotherapy was typically suspended and supportive measures were intensified, with most patients experiencing hematologic recovery sufficient to resume radiotherapy within 3 – 5 days. However, in the non-PEG-rhG-CSF group, one patient developed grade 4 myelosuppression that did not resolve to the required threshold for radiotherapy, even after active intervention, ultimately leading to discontinuation of treatment. In addition, the proportion of patients requiring supplementary rhG-CSF during treatment was 26.67% in the PEG-rhG-CSF group, whereas 83.33% in the non-PEG-rhG-CSF group required additional rhG-CSF administration (**Table 2**). There was no significant difference in the number of patients who completed the extra application of brachytherapy between the PEG-rhG-CSF (n=26) and non-PEG-rhG-CSF (n=23) groups (P > 0.05) (**Table 2**). Collectively, these results demonstrate that PEG-rhG-CSF not only reduces the incidence of severe myelosuppression but also decreases the need for remedial leukocyte-elevating therapy, thereby helping to maintain the continuity and completion rate of radiotherapy.

**Table 2:** Treatment analysis.

	PEG-rhG-CSF (mean ± SD)	Non-PEG-rhG-CSF (mean ± SD)	Statistics	*P*
EQD2 (Gy)	89.98± 2.458	88.77± 2.443	*t* = 1.904	0.062
Radiotherapy Time (weeks)	7.85 ± 0.604	8.86± 0.972	*t* = 4.822	<0.0001
Time to bone marrow suppression (weeks)	4.93 ± 0.22	4.07 ± 0.22	*t* = 2.729	0.008
Concurrent chemotherapy with cisplatin				
Yes	30	30		
No	0	0		
Whether to extend the radiotherapy time			*χ^2^*	0.013
Yes	5 (16.67%)	14 (48.28%)		
No	25 (83.33%)	15 (51.72%)		
Whether to discontinue treatment			*χ^2^*	*>0.9999*
Yes	0 (0%)	1 (3%)		
No	30 (100%)	29 (97%)		
Whether to use rescue rhG-CSF.			*χ^2^*	*<0.0001*
Yes	8 (26.67%)	25 (83.33%)		
No	22 (73.33%)	5 (16.67%)		
Whether to perform the extra application of brachytherapy			*χ^2^*	0.5062
Yes	26 (86.67%)	23 (79.31%)		
No	4 (13.33%)	6 (20.69%)		

EQD2, equivalent biological dose; rhG-CSF, recombinant human granulocyte colony-stimulating factor.

### 3.3 Grade 3/4 ANC and the incidence of FN

**Figure 3:**
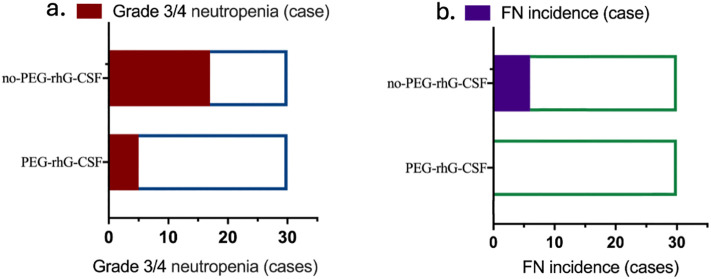
Two horizontal bar charts comparing patients receiving PEG-rhG-CSF versus no PEG-rhG-CSF: **(a)** Fewer cases of grade 3/4 neutropenia are observed in the PEG-rhG-CSF group. **(b)** Fewer cases of FN are observed in the PEG-rhG-CSF group. (FN, febrile neutropenia).

The incidence of grade 3/4 ANC reductions in the PEG-rhG-CSF group was 5 cases (16.67%), compared to 17 cases (56.67%) in the non-PEG-rhG-CSF group, which was significantly lower than that of the non-PEG-rhG-CSF group (*P* = 0.0028) (**Figure 3A**). Similarly, there were no cases of FN in the PEG-rhG-CSF group (0%), while in the non-PEG-rhG-CSF group, six cases of FN were observed—five in week 5 and one in week 8—yielding an incidence of 20%, which was significantly higher than that in the PEG-rhG-CSF group (P = 0.0237) (**Figure 3B**).

### 3.4 Hematological toxicity

**Figure 4:**
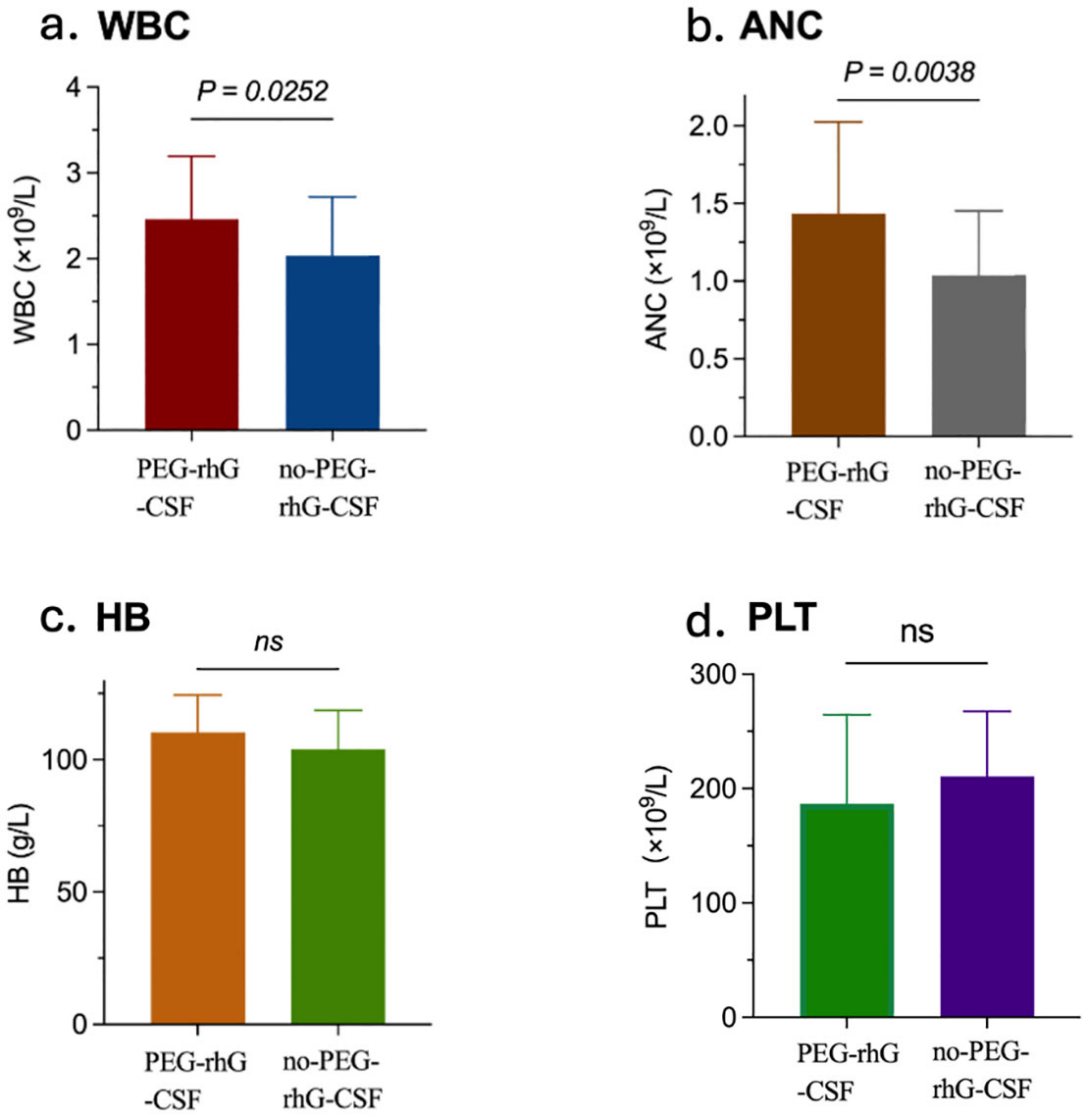
Four bar charts compare outcomes between the PEG-rhG-CSF group and the no PEG-rhG-CSF group during the treatment period: **(a)** WBC is higher in the PEG-rhG-CSF group and is statistically significant (*p*=0.0252). **(b)** ANC is higher in the PEG-rhG-CSF group and is statistically significant (*p*=0.0038). **(c)** HB is comparable between groups; not significant (ns). **(d)** PLT is comparable between groups; not significant (ns). (WBC, white blood cell count; ANC, absolute neutrophil count; HB, hemoglobin level; PLT, platelet count).

According to the World Health Organization (WHO) standards, chemotherapy-induced bone marrow suppression is classified into grades 0 to IV to assess acute and subacute toxic reactions (38). The analysis of hematological toxicity is shown in **Figures 2B** and **4**. During the treatment period, in terms of hematological toxicity, the time to the initial decrease in WBC was 4.93 ± 0.22 weeks in the PEG-rhG-CSF group, while it was 4.07 ± 0.22 weeks in the non-PEG-rhG-CSF group (**Figure 2B**). The difference was statistically significant (*P* < 0.05). **Figure 4**. Analyzing from the lowest values of WBC and ANC, the mean ± SD WBC count in the PEG-rhG-CSF group was 2.46 ± 0.13 × 10^9^/L, while in the non-PEG-rhG-CSF group it was 2.04 ± 0.13 × 10^9^/L. The difference between the two groups was statistically significant (*P* = 0.025). Similarly, the mean ± SD ANC in the PEG-rhG-CSF group was 1.44 ± 0.11 × 10^9^/L, while in the non-PEG-rhG-CSF group it was 1.04 ± 0.08 × 10^9^/L. The difference between the two groups was statistically significant (*P* = 0.004). And also, the mean ± SD HB and PLT was no statistically significant.

### 3.5 Non-hematological adverse reactions

During the treatment period, non-hematologic adverse events primarily consisted of gastrointestinal reactions. Nausea was most commonly observed during the first week of radiotherapy; for most patients, the severity was mild and did not interfere with the continuation of radiotherapy. Prophylactic antiemetic medications were administered during chemotherapy. Diarrhea and increased bowel movements occurred more frequently during weeks 2 to 3. All patients received oral probiotics starting from the initiation of radiotherapy. When gastrointestinal symptoms occurred, symptomatic antidiarrheal medications and adjunctive traditional Chinese medicine therapies were administered promptly, effectively controlling symptoms without any cases leading to treatment interruption or delay. No significant urogenital adverse events affecting the treatment were observed during radiotherapy. Bone pain related to PEG-rhG-CSF could be effectively managed with nonsteroidal anti-inflammatory drugs (NSAIDs). There was no statistically significant difference in the incidence of non-hematologic adverse events between the two groups.

## 4 Discussion

Currently, the preferred treatment regimen for advanced cervical cancer is concurrent chemoradiotherapy combined with brachytherapy (13, 39, 40). This treatment significantly improves tumor local control rates and overall survival through synergistic effects, making it a critical approach for treating advanced cervical cancer. However, this regimen is also associated with significant adverse effects, particularly bone marrow suppression, characterized by a reduction in WBC count and ANC (41). Such bone marrow suppression not only increases the risk of infection but may also lead to interruptions or delays in radiotherapy, prolonging treatment duration and potentially preventing radiotherapy completion within the recommended 8 weeks (42). Delays in radiotherapy have been shown to be closely associated with poor patient prognosis. Therefore, effectively preventing or alleviating bone marrow suppression to ensure the continuity and timeliness of treatment is a pressing clinical challenge.

PEGylated recombinant human granulocyte colony-stimulating factor (PEG-rhG-CSF) is a novel long-acting granulocyte colony-stimulating factor. It exerts its effects primarily by binding to G-CSF receptors on the surface of hematopoietic progenitor cells in the bone marrow, thereby activating multiple intracellular signaling pathways, such as the JAK/STAT and MAPK pathways. This activation promotes the proliferation, differentiation, and maturation of these progenitor cells, accelerating the production and release of neutrophils. In addition to facilitating the release of mature neutrophils from the bone marrow into the peripheral circulation, PEG-rhG-CSF can also prolong neutrophil survival, thereby effectively increasing peripheral white blood cell counts (24, 43). Currently, PEG-rhG-CSF is widely used for the prevention and treatment of chemotherapy-induced neutropenia. During concurrent chemoradiotherapy, PEG-rhG-CSF helps maintain WBC and neutrophil counts (24, 44), alleviates chemotherapy-related bone marrow suppression, reduces treatment interruptions caused by hematological toxicity, and consequently improves radiotherapy completion rates. Studies have shown that timely completion of radiotherapy within 8 weeks is closely associated with improved long-term survival rates in patients (45). Therefore, PEG-rhG-CSF may further improve patient prognosis by ensuring the smooth progression of treatment. However, systematic studies on the impact of PEG-rhG-CSF on radiotherapy completion time during concurrent chemoradiotherapy remain limited, and further exploration of its underlying mechanisms and clinical effects is warranted.

This study conducted a retrospective analysis to evaluate the efficacy and safety of PEG-rhG-CSF in concurrent chemoradiotherapy for cervical cancer. The results showed that 83.33% of patients in the PEG-rhG-CSF group completed radiotherapy within 8 weeks, significantly higher than 51.72% in the non-PEG-rhG-CSF group (*P* < 0.05), indicating that PEG-rhG-CSF effectively improves radiotherapy completion rates. Additionally, the incidence of grade 3/4 neutropenia in the PEG-rhG-CSF group was significantly lower than in the non-PEG-rhG-CSF group (16.67% vs. 56.67%, *P* = 0.003), and no cases of FN were observed in the PEG-rhG-CSF group, compared to 6 cases in the non-PEG-rhG-CSF group (*P* < 0.05). Furthermore, bone marrow suppression occurred later in the PEG-rhG-CSF group than in the non-PEG-rhG-CSF group, and the lowest WBC and ANC levels in the PEG-rhG-CSF group were significantly higher than those in the non-PEG-rhG-CSF group (*P* = 0.025 and *P* = 0.004, respectively), suggesting that PEG-rhG-CSF plays a significant role in mitigating bone marrow suppression. The difference in platelet count changes between the PEG-rhG-CSF group and the non-PEG-rhG-CSF group was not statistically significant. Furthermore, during the entire treatment period, no bleeding events due to thrombocytopenia were observed in the PEG-rhG-CSF treatment group. These findings further confirm the protective effect of PEG-rhG-CSF against hematological toxicity during concurrent chemoradiotherapy.

In terms of safety, this study found that the primary adverse event associated with PEG-rhG-CSF was bone pain (46), which could be alleviated with symptomatic treatment and was well-tolerated by patients without significantly increasing safety risks. This indicates that PEG-rhG-CSF has good tolerability in concurrent chemoradiotherapy for cervical cancer, providing a safe option for clinical application. Moreover, the use of PEG-rhG-CSF not only alleviates chemotherapy-induced neutropenia but may also improve patients’ overall treatment experience and quality of life, enabling them to complete the entire treatment process more smoothly.

Although the results of this study demonstrate the significant advantages of PEG-rhG-CSF in improving radiotherapy completion rates, mitigating bone marrow suppression, and enhancing patient prognosis, there are certain limitations. First, as a retrospective analysis, data collection may be subject to selection bias and information bias, which could affect the reliability of the results. Second, the sample size was relatively small, and the follow-up period was limited, which may not fully reflect the efficacy and safety of PEG-rhG-CSF across different populations. Additionally, this study did not conduct an in-depth analysis of long-term survival outcomes and quality of life, making it difficult to comprehensively evaluate the long-term clinical benefits of PEG-rhG-CSF. Therefore, future large-scale prospective randomized controlled trials are needed to further validate the efficacy and safety of PEG-rhG-CSF and explore its application in different patient subgroups.

In conclusion, PEG-rhG-CSF demonstrates good safety and significant clinical efficacy in concurrent chemoradiotherapy for cervical cancer. By alleviating chemotherapy-induced neutropenia, PEG-rhG-CSF effectively improves radiotherapy completion rates, reduces treatment interruptions, and facilitates timely completion of radiotherapy, thereby improving patient prognosis. This finding has important clinical significance. Further research into the mechanisms and potential applications of PEG-rhG-CSF in cancer treatment may provide new directions for optimizing cervical cancer treatment strategies and ultimately improving patient outcomes and quality of life.

## Data Availability

The original contributions presented in the study are included in the article/supplementary material. Further inquiries can be directed to the corresponding author.
